# Risky health behaviours and chronic conditions among aged persons: analysis of SAGE selected countries

**DOI:** 10.1186/s12877-023-03836-y

**Published:** 2023-03-17

**Authors:** Joseph Kojo Oduro, Joshua Okyere, Jonas Kwame Mawuli Tawiah Nyador

**Affiliations:** 1 Department of Population and Health, University of Cape Coast, Cape Coast, Ghana; 2 Department of Nursing, College of Health Sciences, Kwame Nkrumah University of Science and Technology, Kumasi, Ghana; 3 Department of Mathematics, Universität Hamburg, Hamburg, Germany

**Keywords:** Ageing and aged persons, Chronic conditions, Behavioural change, Health promotion

## Abstract

**Background:**

Increasing trends in risky health behaviours contribute to chronic health problems among the rapidly growing ageing population. Therefore, we examined the association between risky health behaviours and chronic health conditions among persons 50 years and older.

**Methods:**

This study was a secondary analysis of longitudinal survey data from the 2007 Study on Global Ageing and Adult Health (SAGE Wave 1) conducted by the World Health Organization. Multilevel logistic regression techniques were used to examine high social cohesion among the aged. The output was reported as odds ratios (OR) and adjusted odds ratios (aOR).

**Results:**

Generally, the level of chronic conditions was 81.5% for all countries. Older adults in Ghana had the highest chronic conditions (94.0%) while the Russian Federation recorded the lowest (58.6%). The risk of chronic conditions was higher among the oldest-old (OR = 1.70, 95% CI = 1.29, 2.25), those who smoke tobacco (OR = 1.13, 95% CI = 1.01, 1.25) or drink alcohol (OR = 1.17, 95% CI = 1.06,1.29), and among those who live in rural areas (OR = 1.31, 95% CI = 1.16, 1.49). However, the odds were lower among females (OR = 0.88, 95% CI = 0.69,0.85), and those who were not working (OR = 0.52, 95% CI = 0.47, 0.58).

**Conclusion:**

We conclude that it is important to improve the health status of older people. To achieve this, there must be interventions and policies to facilitate the adoption of healthy or physically active lifestyles among older people. This could be achieved by strengthening advocacy and health education about the dangers of living a sedentary lifestyle, consuming alcohol and tobacco. Whatever behavioural change interventions, advocacy and health education must target high-risk sub-populations including the oldest-old, and those with low economic status. Given the regional disparities identified, it is necessary to prioritise older people residing in rural areas. The study underscores a need to provide more primary healthcare facilities in the rural areas of the countries included in this study. Such an initiative is likely to increase accessibility to healthcare services and information that would impact positively on the lifestyle behaviours of older people.

## Background

Risky health behaviour is a conceptual topic of great importance to both the medical and the non-medical community due to the rapid rise in the ageing population since 1950 [1]. This trend was followed by increased risky health behaviours among the ageing population. Meanwhile, risky health behaviour is usually described fundamentally as the human behaviours and choices of life that potentially impact health negatively. Such behaviours include excessive alcohol consumption, smoking, non-engagement in physical activities, non-prescribed use of medicine, substance use and abuse, such as cocaine and risky sexual behaviours such as engaging in unprotected sexual intercourse and multiple sexual partnerships [2, 3]. These risky health behaviours may further be exacerbated by other social determinants of health (i.e., education level, place of residence, income or wealth status, and employment status) to affect the health outcomes of aged persons. Hence, the need to explore the association between risky health behaviours and chronic health conditions of the aged.

Studies from several countries have uncovered different risky health behaviours among aged persons. An increase in alcohol consumption among the aged 65 years or over, was found in Sweden [2], the Netherlands and Germany [4]. Other studies clearly showed a significant association between alcohol consumption among the aged and some socio-demographic factors like sex, age, attainment of higher education, not living alone, not being depressed [4–7], social isolation [8, 9], and living arrangements [7, 10]. For instance, a study based on the Chinese Longitudinal Healthy Longevity Survey showed that the odds of alcohol consumption were significantly lower among both older men, and women living with both a spouse and children, compared with those living alone [7]. Relatedly, a study from Thailand showed that male Thai adults aged 50 years and older were more likely to consume alcohol compared to females [5]. Similarly, a study conducted among South African older adults revealed that being male was associated with high odds of risky drinking compared to female older adults [6].

On the other hand, tobacco usage is among the avoidable risky health behaviours, yet it is a leading cause of death among the aged [11, 12]. Studies on tobacco usage in several countries have shown a total 11.5–13% prevalence of tobacco use among the aged [13]. There was a prevalence of 15.3% and 8.6% among men and women, respectively. A higher risk of dementia and mortality rate among aged persons have also been found among the smoking-aged population as compared to non-smokers in this cohort [13, 14]. The same study also showed a positive association between smoking status and lower educational attainment [14]. A systematic review has also shown that the risk of stroke increased by 12% for each increment of 5 cigarettes per day [15]. Leone [16] has also reported that tobacco smoking exacerbates the risk of hypertension which can further increase the odds of developing cardiovascular conditions. An Iranian study conducted showed that smoking was significantly higher among men living alone and having heart disease [17].

Physical inactivity (lack of physical activeness) is the fourth leading high-risk factor for global mortality [18, 19]. Approximately 21–25% of breast and colon cancers, 27% of diabetes and 30% of ischaemic heart disease were due to physical in-activeness [20], and consequently, a reduction in the quality of life of aged persons [21]. A study carried out in Shanghai, showed that 83.3% of the aged living alone did not perform any adequate physical activity per week [22]. According to a related study, chronic conditions (e.g., stroke, Parkinson’s disease, hip fracture) among the aged population increases the risk of disability which may lead to a large impact on the decline in function among the aged population [23].

Increasing trends in risky health behaviours are contributing factors to chronic health problems among the rapidly growing ageing population [24]. Health behaviour risks such as smoking, alcohol consumption, and physical inactivity can have long-lasting health implications, which turn to negatively influence health in older age [7]. Understanding the relationship between risky health behaviours and chronic health conditions among aged persons is essential to developing interventional programs to improve their health status. However, there is a dearth of evidence for multi-country analysis on the subject in question. To the best of our knowledge, no study has assessed how risky health behaviour is associated with the risk of developing chronic conditions across different countries and from different continents. We bridge this knowledge gap by including six countries from Asia, Africa and Eastern Europe. The study aimed to examine the association between risky health behaviours and chronic health conditions among aged persons.

## Methods

### Data source

**Fig. 1:**
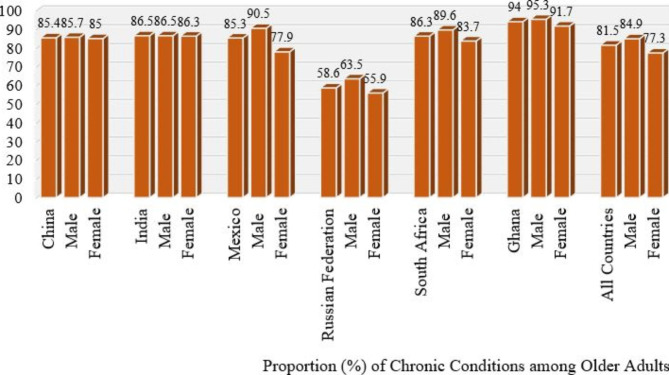
Chronic Conditions of Older Adults by Country

This study was a secondary analysis of longitudinal survey data of the 2007 Study on Global Ageing and Adult Health (SAGE Wave 1) conducted by the World Health Organization (WHO) for six countries (see Fig. 1). SAGE Wave 1 provides a comprehensive dataset on the health and well-being of aged 18–49 years and 50 years and above. SAGE is a nationally representative sample survey collected over a five-year period in six countries including China, Ghana, India, Mexico, Russian Federation, and South Africa. The survey focuses on the health and wellbeing as well as risky health behaviours and chronic health conditions of young (18–49 years) and older (50+) adults. SAGE surveys follow the standard procedures (i.e., sampling, questionnaire development, data collection, cleaning, coding and analysis) which allow for cross-country comparison. The survey employs a stratified two-stage sampling technique. The initial stage involves the selection of Primary Sampling Units (PSUs) by region and location (urban/rural) across the six countries. The second stage involved the systematic selection of enumeration areas (EAs), households (HH) with 50 + adults, and households (HH) with ages 18–49. Therefore, the population size for the SAGE selected countries (China = 15,050, Ghana = 5,573, India = 12,198, Mexico = 5,448, Russian Federation = 4,947, and South Africa = 4,227) was 47,443. For this study, only the aged 50 and over (n = 19,561) who had complete information on the variables of interest were included. Respondents were classified into four categories of functional age brackets: the “younger old” (50–64 years) “young old” (65–74 years); the “old-old” (75–84 years); and the “oldest old” (85 years and above). The data was deemed suitable for this study because it is nationally representative.

### Study variables

#### Response variables

The response variable used in this study was chronic conditions. In this study, chronic conditions were defined as conditions that last more than a year and demand continuing medical attention and limit activities of daily living among the aged. It was derived from set of questions; (1) Have you ever been diagnosed with/told you have arthritis? (2) “Have you ever been told by a health professional that you have had a stroke? (3) Have you ever been diagnosed with angina or angina pectoris (a heart disease)? (4) Have you ever been diagnosed with diabetes (high blood sugar)? (5) Have you ever been diagnosed with chronic lung disease (emphysema, bronchitis, COPD)? (6) Have you ever been diagnosed with asthma (an allergic respiratory disease)? (7) Have you ever been diagnosed with depression? And lastly, (8) Have you ever been diagnosed with high blood pressure (hypertension)? The response categories to these questions were dichotomous “1 = Yes”, and ”2 = No”. Principal Component Analysis (PCA) was used to create an index for high and low chronic conditions.

In all, eight conditions including arthritis, stroke, angina, diabetes, lung disease, asthma, depression, and hypertension. To examine the predictors of chronic conditions of the aged, factor analysis was used to derive a composite indicator to measure the overall chronic condition of the aged. This technique was adopted because it addresses the problem of multicollinearity and assigns indicator weights based on the variations in the responses. Factor analysis involves a mathematical procedure that transforms several correlated variables into a smaller number of uncorrelated variables called factor scores (indices). The factor scores are a linear combination of the original variables which are derived in decreasing order of importance with the first factor score accounting for as much of the variability in the data as possible, and each succeeding factor accounting for as much of the remaining variability. Suppose there are p variables, X1, X2 … Xp, the first factor score (Z1) is linear combination of these variables, given by.

Z_1 = a_11 X_1 + a_12 X_2+⋯+a_1p X_p

where the coefficients a_11,a_12,…a_1p are the factor loadings (weights) used to derive the factor scores, chosen such that the variance of Z_1 is maximised. Indicators assigned higher factor loadings contribute more to the factor scores (composite index).

Table 1 shows the factor loadings for the first-factor score. The factor loadings for all the indicators were indicative of positive chronic conditions suggesting that the first-factor score represents the overall chronic condition of the aged. To identify the factors associated with high chronic condition, the first factor scores (composite index) was ranked and the top 40.0% coded as “1” to represent those with high chronic conditions and “0” to represent those with low chronic conditions. The eight items and specific indicators used to quantify the chronic condition of the aged are shown in Table 1.

**Table 1:** Chronic Conditions, Indicator and Factor Scores

Chronic condition	Indicator	Factor score
Arthritis	I have arthritis	0.51
Stroke	I have stroke	0.37
Angina	I have angina	0.64
Diabetes	I have diabetes	0.33
Lung disease	I have lung disease	0.42
Asthma	I have asthma	0.27
Depression	I have depression	0.27
Hypertension	I have hypertension	0.65

### Explanatory variables

Ten explanatory variables that were considered in this study include the main (tobacco use, alcohol use and physical activity) as well as residence, sex, age, marital status, education, work status, and income quantile. During the survey, the key explanatory variables were measured by the questions; (1) Have you ever smoked tobacco or used smokeless tobacco? (Tobacco use), (2) Have you ever consumed a drink that contains alcohol (such as beer, wine, spirits, etc.)? (3) Does your work involve moderate-vigorous activity that causes large increases in breathing or heart rate, [like heavy lifting, digging or chopping wood] for at least 10 min continuously? or (4) Do you walk or use a bicycle (pedal cycle) for at least 10 min continuously to get to and from places? Responses for these were “1 = Yes”, and ”2 = No”. A “Yes” response to 3.) and 4.) indicated positive physical activities while a “No” suggested otherwise. Apart from the key explanatory variables, seven additional variables were included. These are residence, sex, age, marital status, education, work status, and income quintile. The following variables were recoded to suit the analysis: marital status was recorded as “Never married = 0”, “currently married/cohabiting = 1”, “separated/divorced = 2” and “Widowed = 3”; education into “No formal education = 0” “primary = 1”, “Secondary = 3” and “tertiary = 3; work status was recoded into “Yes, working = 1”, “Not working = 0”, and lastly, income coded into five quintile; “Lowest = 0”, “Low = 1”, “Moderate = 2”, “High = 3”, “Highest = 4”.

### Data analysis

We conducted both descriptive and inferential analyses. At the descriptive level, we analysed the background characteristics of the aged (univariate). We also computed the proportion of the aged with high chronic conditions by background characteristics (bivariate). Chi-square tests were used to investigate significant differences (p < 0.05). Afterward, multilevel binary logistic regression techniques were used to examine high chronic conditions among older adults. A two-level (individuals nested within communities) multilevel binary logistic regression was used to examine the predictors of high chronic conditions among older adults. Model 0 is a null model which did not account for any of the predictors. Model 1 included the SAGE selected countries by high chronic conditions among older persons to account for some of the survey weights. Model 2 added the primary variables (tobacco use, alcohol use and physical activity), while Model 3 was a complete model accounting for all the important confounding variables to assess their combined association with the outcome variable (high chronic conditions). The output was reported as Odds Ratios (ORs) and Adjusted Odds Ratios (AORs) for Model 1, 2, and 3 respectively at a 95% confidence interval. A test for multicollinearity was conducted and each value of the variance inflation factor (VIF) was below 5.00. Thus, the assumption of multicollinearity was met. All frequency distributions were weighted to adjust for the complex sampling structure of the data in the regression analyses. The entire analysis was carried out using SPSS version 25 and R for Windows.

### Ethical approval

All methods were carried out in accordance with relevant guidelines and regulations. The study conforms to the Helsinki Declaration and Belmont Declaration. WHO- Ghana SAGE wave 1 study was approved by the Ethics Review Committee, World Health Organization, Geneva, Switzerland, and the Ghana Health Service. Written informed consent was given by all individuals. The authors of this manuscript were not directly involved in the data collection processes but rather obtained access by requesting the data. The dataset can be accessed at https://apps.who.int/healthinfo/systems/surveydata/index.php/catalog/sage.

## Results

Results in Fig. 1 show the level of chronic conditions of older adults among the six selected countries by sex. Generally, the level of chronic conditions was 81.5% for all countries. In all countries, males (84.9%) had higher chronic conditions than females (77.3%). Older adults in Ghana had the highest chronic conditions (94.0%) with males (95.3%) having higher chronic conditions than their female (91.7%) counterparts while the Russian Federation recorded the lowest (58.6%) with their males having higher chronic conditions (63.5%) when compared to the females (55.9%).

Table 2 shows the results of the univariate analysis. More than half (61.0%) of the older adults reside in the urban areas and are males (55.5%) within the ages 50–64 years (62.7%). Seven out of ten were married/cohabiting (73.9%) while about five out of ten had secondary level of education (46.2%). A little more than half of the older adults were not working (54.3%). Furthermore, a higher proportion of the older adults were in the highest income quintile (26.1%). More than half of the older adults do not smoke or drink but do moderate-vigorous activity/walk/bike (60.9%, 54.9% and 69.5%) respectively. Meanwhile, most of them (81.5%), had chronic conditions.

**Table 2:** Background characteristics of respondents (n = 19,561)

Background characteristics	Sample size	Percent (%)
Place of residence		
Urban	11,929	61.0
Rural	7632	39.0
Gender		
Male	10,847	55.5
Female	8714	44.5
Age		
50–64	12,266	62.7
65–74	4962	25.4
75–84	2016	10.3
85+	317	1.6
Marital status		
Not married	606	3.1
Currently Married/Cohabiting	14,730	75.3
Separated/divorced	994	5.1
Widowed	3231	16.5
Education		
No formal education	23	0.1
Primary	8508	43.5
Secondary/High school	9045	46.2
Tertiary	1985	10.1
Work status		
Working	8931	45.7
Not working	10,630	54.3
Income		
Lowest	2621	13.4
Low	3347	17.1
Moderate	3874	19.8
High	4606	23.5
Highest	5113	26.1
Tobacco use		
Yes	7655	39.1
No	11,906	60.9
Alcohol consumption		
Yes	8824	45.1
No	10,737	54.9
Physical activities		
Yes	13,587	69.5
No	5974	30.5
Chronic Conditions		
No Chronic Conditions	3624	18.5
Chronic Conditions	15,937	81.5

In Table 3, the results of high chronic conditions and background characteristics of older adults are presented. Generally, majority of the older adults had chronic conditions (81.5%) with the highest incidence of chronic conditions recorded in Ghana (94.0%). Results show that living in rural areas (87.7%) and being a male (84.9%) were associated with high chronic conditions. About nine out of ten of the older adults who are younger-olds (50–64 years) (87.5%) had chronic conditions when compared with the old-olds (75–84 years) (62.7%). Moreover, those who are married/cohabiting (83.9%) and had no formal education (91.3%) had higher proportions of chronic conditions. Furthermore, those who are working (90.2%) and with highest income (83.5%) were associated with high chronic conditions.

**Table 3:** Distribution of Chronic Conditions by Background Characteristics (n = 19,561)

	High Chronic Conditions		
**Background characteristics**	**%**	**(n) 95**% **CI**	**P-value**
Overall	81.5	(15,937) [0.81, 0.82]	< 0.001
Countries			
China	85.4	7317 [0.85, 0.86]	
India	86.5	2120 [0.85, 0.88]	
Mexico	85.3	848 [0.83, 0.88]	
Russian Federation	58.6	2129 [0.57, 0.61]	
South Africa	86.3	1732 [0.85, 0.88]	
Ghana	94.0	1791 [0.93, 0.95]	
Place of residence			< 0.001
Urban	77.5	(9246) [0.77, 0.78]	
Rural	87.7	(6691) [0.87, 0.88]	
Sex			< 0.001
Male	84.9	(9205) [0.84, 0.86]	
Female	77.3	(6732) [0.76, 0.78]	
Age			< 0.001
50–64	87.5	(10,728) [0.87, 0.88]	
65–74	75.1	(3724) [0.74, 0.76]	
75–84	62.7	(1264) [0.60, 0.65]	
85+	69.7	(221) [0.64, 0.76]	
Marital status			< 0.001
Not married	83.0	(606) [0.80, 0.86]	
Currently Married/Cohabiting	83.9	(14,730) [0.83, 0.85]	
Separated/divorced	81.4	(994) [0.79, 0.83]	
Widowed	70.3	(3231) [0.68, 0.72]	
Education			< 0.001
No formal education	91.3	(21) [0.79, 1.03]	
Primary	85.2	(7246) [0.84, 0.86]	
Secondary/High school	78.8	(7130) [0.78, 0.80]	
Tertiary	77.6	(1540) [0.76, 0.80]	
Work status			< 0.001
Working	90.2	(8059) [0.90, 0.91]	
Not working	74.1	(7878) [0.73, 0.75]	
Income			< 0.001
Lowest	79.9	(2094) [0.78, 0.82]	
Low	80.0	(2677) [0.78, 0.82]	
Moderate	79.8	(3093) [0.78, 0.81]	
High	82.5	(3802) [0.81, 0.84]	
Highest	83.5	(4271) [0.82, 0.85]	
Tobacco use			< 0.001
Yes	83.4	6387 [0.82, 0.84]	
No	80.2	9550 [0.79, 0.81]	
Alcohol consumption			< 0.001
Yes	79.0	6971 [0.78, 0.80]	
No	83.5	8965 [0.83, 0.84]	
Physical activities			< 0.001
Yes	83.0	11,276 [0.82, 0.84]	
No	78.0	4661 [0.77, 0.79]	

A sequential modelling approach was used to examine the association between risky health behaviours and chronic health conditions and background characteristics that were predictors of high chronic conditions among older adults. Model 1 included the country of origin to account for some of the survey weights. Model II added the risky health behaviours, while Model III included the background characteristics. A two-level (individuals nested within countries) multilevel binary logistic regression was used to examine the predictors of high chronic conditions among older adults.

Table 4 shows the estimated odds ratios of high chronic conditions, along with their corresponding 95% confidence intervals and model summary statistics. The variance terms presented in Table 4 are significant at the community level. Model I reveals that there are significant differences between countries with respect to experience of high chronic conditions. The variance of the random effects at the community level was 43.8% of the between country variations in high chronic conditions with respect to older adults and risky health behaviours and was statistically significant at p < 0.01. When the primary factors were introduced in model II, the variance of the random effects was reduced by 0.68%. Meanwhile, the variance was still significant. Introduction of the confounding factors in model III, showed a further reduction in the variance terms by 0.35% but the variance was still significant at p < 0.01. This shows that significant differences exist between countries/communities with regards to the proportion of the older adults with high chronic conditions. Further analysis in Table 3 shows that the estimated AIC (Akaike information criterion) for Model I was 17059.7. The introduction of the primary factors (countries) led to further reduction of the AIC from 17059.7 to 16995.9 in Model II. The AIC decreased again from 16995.9 to 16240.4 after the confounders were added in Model III. Thus, the lower the AIC, the better the model and the closer it is to the unknown population model [25, 26].

Results in Table 4 revealed that country of origin, tobacco use, alcohol consumption, physical activities, place of residence, sex, age, work status and income of older adults have significant impact on their chronic conditions. Interpretation of the model results is based on the final model (Model III). The odds of having chronic conditions were higher among older adults in Ghana (OR = 2.27; 95%CI = 1.77, 2.92) compared to those from China. With respect to the risky health behaviours, older adults who smoke or drink alcohol, were more likely to experience high chronic conditions (OR = 1.13; 95%CI = 1.01, 1.25), (OR = 1.17; 95%CI = 1.06,1.29) when compared to those who do not smoke or drink (Model III). On the other hand, older persons who engaged in physical activities (moderate-vigorous activities/walk/bike) had lower odds of experiencing chronic conditions (OR = 0.81; 95%CI = 0.75, 0.89) when compared to those who do not engage in any physical activities.

Furthermore, residing in the rural area was associated with high odds of experiencing high chronic conditions (OR = 1.31, 95% CI = 1.16, 1.49) when compared with living in the urban area. Older adults who were females were less likely to experience high chronic conditions (OR = 0.88; 95%CI = 0.69,0.85) when compared with their male counterparts. Concerning ages of respondents, oldest-olds (85 + years) had higher odds of high chronic conditions (OR = 1.70; 95%CI = 1.29, 2.25) when compared with the younger-olds (50–64 years). Older adults who were not working were less likely to experience high chronic conditions (OR = 0.52; 95%CI = 0.47, 0.58) as compared to their counterparts who were working. In furtherance, older adults with moderate income were less likely to have chronic conditions (OR = 0.95; 95%CI = 0.82, 1.10) as compared to those with the lowest income (Table 4). Education did not show a statistically significant result in this study, hence, it was not included in the presentation of the result in Table 4.

**Table 4:** Estimated Odds Ratios of Association between Risky Behaviours and chronic conditions (n = 19,561)

BACKGROUND CHARACTERISTICS	Model I	Model II	Model III
	OR [95% CI]	OR [95% CI]	OR [95% CI]
Countries			
China	1.00	1.00	1.00
India	1.17 [0.96,1.43]	1.11 [0.91,1.35]	0.84 [0.70,1.02]
Mexico	1.04 [0.81,1.33]	1.07 [0.84,1.37]	1.27 [1.00,1.62]*
Russian Federation	0.23 [0.20,0.26] ***	0.24 [0.21,0.27] ***	0.30 [0.27,0.35] ***
South Africa	1.12 [0.91,1.37]	1.26 [1.02,1.55]	1.35 [1.11,1.65] **
Ghana	2.94 [2.28,3.78] ***	2.99 [2.32,3.86] ***	2.27 [1.77,2.92] ***
**PRIMARY FACTORS**			
Risk behaviours			
Tobacco use			
No		1.00	**1.00**
Yes		0.91 [0.83,1.00]*	1.13 [1.01,1.25]*
Alcohol use			
No		1.00	1.00
Yes		1.05 [0.96,1.16]	1.17 [1.06,1.29]**
Physical activity			
No		1.00	1.00
Yes		0.70 [0.64,0.76] ***	0.81 [0.75,0.89]***
**CONFOUNDING FACTORS**			
**Individual level factors**			
Place of residence			
Urban			1.00
Rural			1.31 [1.16,1.49] ***
Sex			
Male			1.00
Female			0.88 [0.69,0.95] ***
Age			
50–64			1.00
65–74			0.56 [0.51,0.62] ***
75–84			0.40 [0.35,0.45] ***
85+			1.70 [1.29,2.25] *
Work status			
Working			1.00
Not Working			0.52 [0.47,0.58] ***
Income			
Lowest			1.00
Low			0.88 [0.76,1.02]
Moderate			0.83 [0.72,0.96] *
High			0.89 [0.77,1.03]
Highest			0.95 [0.82,1.10]
Variance of the random Effects [SE]			
Community	0.3132 [0.02]**,	0.311 [0.02]**	0.205 [0.01]**,
% ∆ in random effect	4.38	-0.68	-0.35
Community			
Deviance	17045.7	16975.9	16194.4
AIC	17059.7	16995.9	16240.4

P < 0.001***, p < 0.01**, p < 0.05*

## Discussion

The present study sought to examine the association between risky health behaviours and reported chronic conditions among older people across six countries. Overall, the level of chronic conditions was 85.1%, indicating the pervasiveness of chronic conditions among older people. Ghana had the highest level of chronic conditions while the Russian Federation reported the least prevalence. These between-country differences in the prevalence of chronic conditions may be a reflection of existing healthcare disparities which tend to be more profound in developing countries such as Ghana. Furthermore, the wide difference observed between Ghana and the Russian Federation could be attributed to the varied economic disparity of the SAGE participating countries [27]. Ghana has the least GDP per capita among the six participating SAGE countries, whereas the Russian Federation had the highest GDP [27]. This reflects a situation that creates significant inaccessibility to healthcare services for older people in Ghana.

A major finding from our study was that tobacco use was significantly associated with self-reported chronic conditions, as older people who used tobacco were more likely to report at least one chronic condition compared to those who did not use tobacco. The result mirrors that of previous studies conducted in Ghana [28] and India [29]. A related study revealed that tobacco use among older people has the tendency to increase blood sugar level as well as facilitate insulin resistance [30]. Analogously, Shah and Cole [31] also revealed a dose-response relationship between tobacco use and the risk of stroke. Therefore, the more an individual smoke, the higher their risk of developing a stroke. Similarly, evidence from the Global Burden of Disease has also shown significant association between tobacco smoking and the risk of developing chronic obstructive pulmonary disease (COPD) and lung diseases [32]. Thus, corroborating our observation that tobacco use is associated with a higher risk of chronic conditions among older people.

Consistent with previous studies [29, 30], we found that older people who consumed alcohol were 1.17 times more likely than those who did not consume alcohol to report at least one chronic condition. Our observation is further supported by the findings of a systematic review which showed that alcohol consumption was associated with an increased risk of hypertension [33]. A plausible explanation for this observation could be due to the fact that alcohol consumption in the absence of physical activity or exercise exacerbates the risk of obesity which is a known stronger predictor of chronic conditions in older people [34]. Therefore, the findings suggest that reducing alcohol consumption in old age may be a protective mechanism against chronic conditions including angina, hypertension, arthritis, diabetes and stroke.

The study also indicates that physical activity, captured as walking/biking, is significantly associated with reported chronic conditions among older people. From the findings, compared to older people who did not engage in walking or biking, those who engaged in these physical activities were less likely to report a chronic condition. Similar findings have been reported by previous studies [35, 36]. A sedentary lifestyle increases the tendency of abdominal obesity which exerts substantial pressure on the body’s metabolism and blood flow [37]. This exacerbates the risk of hypertension among older people. Given that hypertension is a risk factor for diabetes and stroke, physical inactivity among older people may result in a vicious cycle of chronic diseases [38]. Thus, reaffirming the essentiality of maintaining physical activity in old age.

Beyond the main findings that there is a significant association between risky health behaviours and self-reported chronic conditions among older people, we found a significant association between confounding variables (place of residence, sex, age, work status and income) and chronic conditions. Older people with moderate income were less likely to report chronic diseases compared to those in the lowest income status, a result that is in agreement with a previous study [39]. Often, foods that are high in added sugar and fats are the least expensive compared to whole grains and other healthy food choices [40]. As such, old people with a better economic status can afford much healthier food choices, thereby reducing their risk of chronic conditions. Another possible explanation could be that, unlike individuals from low income households, older people from moderate to high income households have greater access to healthcare services [41]. This makes them more likely to be exposed to preventive health messages that could nudge them into engaging in less risky health behaviour; hence, reducing their risk of chronic diseases. Closely related to income status, we found a lower likelihood of reporting chronic conditions among persons who were not working as compared to those working. This finding may be explained from the perspective that older people who are not working may not be exposed to high level of stress that aggravates from work demands; thus, serving as a protective factor against stress-related ill health outcomes that have the potential of transitioning into chronic conditions such as hypertension and depression.

From our findings, the oldest-olds (85 + years) had higher odds of chronic conditions compared to the younger-olds. A similar finding was reported in China [42] where the odds of having chronic diseases intensified after age 80. Plausibly, we can explain this from the perspective that, the biological system of older people becomes highly compromised at the stage of oldest-old, which makes them more susceptible to disease conditions. Also, results indicate that females were less likely to report chronic conditions compared to older males. Several reasons may explain this observation. The findings can be explained from two main perspectives: the biological and behavioural perspectives. From a biological perspective, previous studies have shown that sex hormones and chromosomal differences serve as protective factors against chronic conditions like hypertension among women compared to men [43]. From a behavioural perspective, the findings may be explained by the differences in the risk-taking attitudes of males and females. For instance, compared to males, females are less likely to engage in risky health behaviours such as alcohol consumption and tobacco use, often due to the low social acceptability of such behaviours among females [44]. Hence, reducing their risk of reporting chronic conditions.

Unexpectedly, our findings revealed that residing in rural areas significantly increased the odds of reporting chronic conditions. This observation is incongruent with previous studies that have found lower odds of having chronic diseases such as diabetes and high blood pressure among older people in rural residences [42, 45]. Notwithstanding, the result is in agreement with a related study from China that found the odds of depression to be significantly high among rural dwelling older people [46]. The observed rural-urban disparities in the risk of having chronic conditions may be explained from the perspective that rural dwelling older adults may lack health-related information and knowledge, and individuals in rural areas may have less education than adults in urban areas, which may make them less aware of or skeptical of efforts to prevent chronic diseases [47]. Another possible explanation for the higher odds of reporting chronic conditions in rural areas could be due to inadequate levels of basic infrastructure in rural areas, notably in regard to the home heating, water supply, and transportation systems [48].

## Limitations

The study has some limitations. First, data on chronic disease in the SAGE data was self-reported. As such, there is the possibility of recall bias and social desirability bias. Also, due to the self-reported chronic diseases, there is the possibility of underreported prevalence. We are unable to establish causality between risky health behaviours and having chronic conditions due to the cross-sectional study design adopted in the SAGE data. For establishing causal relationships, there need to be a longitudinal study. Nevertheless, the study has some strengths worthy of acknowledgment. The use of nationally representative population-based survey data to examine the association between risky health behaviours and having chronic conditions is one of the study’s strengths.

## Conclusion

We conclude that it is important to improve the health status of older people. To achieve this, there must be interventions and policies to facilitate the adoption of healthy or physically active lifestyles among older people. This could be achieved by strengthening advocacy and health education about the dangers of living a sedentary lifestyle, consuming alcohol and tobacco. Whatever behavioural change interventions, advocacy and health education must target high-risk sub-populations including the oldest-old, and those with low economic status. Given the regional disparities identified, it is necessary to prioritise older people residing in rural areas. The study underscores a need to provide more primary healthcare facilities in the rural areas of the countries included in this study. Such an initiative is likely to increase accessibility to healthcare services and information that would impact positively on the lifestyle behaviours of older people.

## Data Availability

The datasets analysed during the current study are freely available at: https://apps.who.int/healthinfo/systems/surveydata/index.php/catalog/sage.

## Abbreviations

EAs: Enumeration Areas
PCA: Principal Component Analysis
PSUs: Primary Sampling Units
SAGE: Study on Global Ageing and Adult Health
WHO: World Health Organization
